# Characteristics of the Mesophotic Megabenthic Assemblages of the Vercelli Seamount (North Tyrrhenian Sea)

**DOI:** 10.1371/journal.pone.0016357

**Published:** 2011-02-03

**Authors:** Marzia Bo, Marco Bertolino, Mireno Borghini, Michela Castellano, Anabella Covazzi Harriague, Cristina Gioia Di Camillo, GianPietro Gasparini, Cristina Misic, Paolo Povero, Antonio Pusceddu, Katrin Schroeder, Giorgio Bavestrello

**Affiliations:** 1 DisMar, Università Politecnica delle Marche, Ancona, Italy; 2 DipTeRis, Università degli Studi di Genova, Genova, Italy; 3 CNR-ISMAR, Sezione di La Spezia, Pozzuolo di Lerici, Italy; National Oceanic and Atmospheric Administration/National Marine Fisheries Service/Southwest Fisheries Science Center, United States of America

## Abstract

The biodiversity of the megabenthic assemblages of the mesophotic zone of a Tyrrhenian seamount (Vercelli Seamount) is described using Remotely Operated Vehicle (ROV) video imaging from 100 m depth to the top of the mount around 61 m depth. This pinnacle hosts a rich coralligenous community characterized by three different assemblages: (i) the top shows a dense covering of the kelp *Laminaria rodriguezii*; (ii) the southern side biocoenosis is mainly dominated by the octocorals *Paramuricea clavata* and *Eunicella cavolinii*; while (iii) the northern side of the seamount assemblage is colonized by active filter-feeding organisms such as sponges (sometimes covering 100% of the surface) with numerous colonies of the ascidian *Diazona violacea*, and the polychaete *Sabella pavonina*. This study highlights, also for a Mediterranean seamount, the potential role of an isolated rocky peak penetrating the euphotic zone, to work as an aggregating structure, hosting abundant benthic communities dominated by suspension feeders, whose distribution may vary in accordance to the geomorphology of the area and the different local hydrodynamic conditions.

## Introduction

Seamounts are major topographic features of all ocean basins and are defined as undersea mountains rising more than 100 m from the seafloor without breaking the sea surface [1].

An abrupt topographic elevation, such as a seamount, over a flat deep bottom produces profound effects on the surrounding physical environment (in the form of upwellings, turbulence, Taylor cones, eddies, …) and, in turn, on the local dynamics of plankton and benthos [2], [3], [4]. In particular the establishment of megabenthic assemblages on seamounts (mainly composed of suspension feeders) may be affected by several environmental conditions [5], for example the presence of steep slopes, which reduce the spatial extent and enhance the coexistence of communities with different bathymetric requirements [6], [7]. Also the depth of the peak, its geomorphology and the geographic isolation of the mount are important, since they influence the biocoenoses composition [2], [8]. The role of temperature and pressure have also been discussed as factors controlling benthos zonation [9]. However the distribution of suspension feeders seems to be mainly related to the current regime. Hydrodynamism in fact may affect sedimentation rates, larval settlement and, together with localized vertical nutrient fluxes and material retention, the productivity of the area [6], [9]–[12].

The Mediterranean Sea hosts about 1% of the total predicted large seamounts identified for the world's major basins [13]. These underwater mountains have been relatively well investigated from the paleo-geological point of view [e.g. 14]–[18], but very few data are available concerning the composition of their megabenthic assemblages [19]–[20]. For example a rich algal community and the mollusc fauna were described on the top (from 20 m depth) of the Amendolara Bank in the Ionian Sea [21]–[24]. The benthic fauna of the summit of the Eratosthenes Seamount (756 m depth) to the south of Cyprus, in the Eastern Mediterranean, was also studied [19]. Some biological data were provided by geological surveys made on two shallow mounts (summits at less than 100 m depth) in the Aegean Sea [25], [26]. A similar geomorphological survey with submarine images was made on the Vercelli Seamount [19], while a detailed ROV imaging campaign was used to describe the coralligenous assemblages from 80 to 170 m depth on four shallow seamounts along the Spanish coast [27].

**Figure 1:**
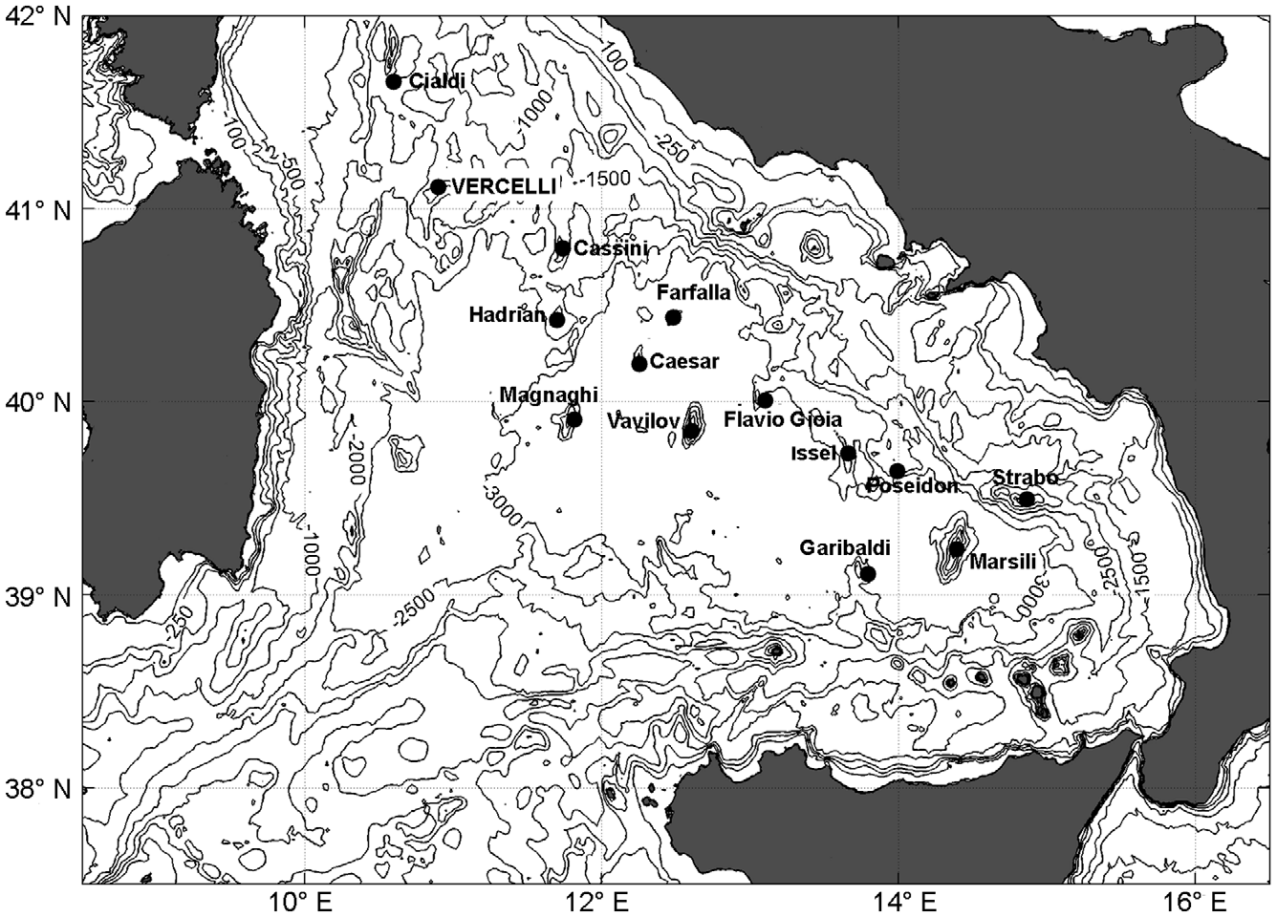
Location of the Tyrrhenian major seamounts.

The Tyrrhenian bathyal plain is spotted by at least 14 large and intermediate seamounts (Fig. 1). Differently to all other structures, Vercelli Seamount (together with Strabo Seamount), has a shallow peak (60–70 m depth) where there is sufficient light for photosynthetic communities to develop. This structure therefore is a good opportunity to study the characteristics of a Mediterranean coralligenous ecosystem, until now mainly considered in coastal areas, on an isolated topographic feature, subjected to peculiar hydrodynamic conditions and spatial limitations.

The aim of this paper is to describe the megabenthic biodiversity of the mesophotic zone of the Vercelli Seamount (60–100 m) and to compare it with existing data concerning both other shallow seamounts and continental areas. Moreover this study aims to give account of the peculiar structure of the Vercelli's community distinguished into various assemblages defined by local hydrodynamic, geomorphologic and trophic characteristics.

## Materials and Methods

### Study area

**Figure 2:**
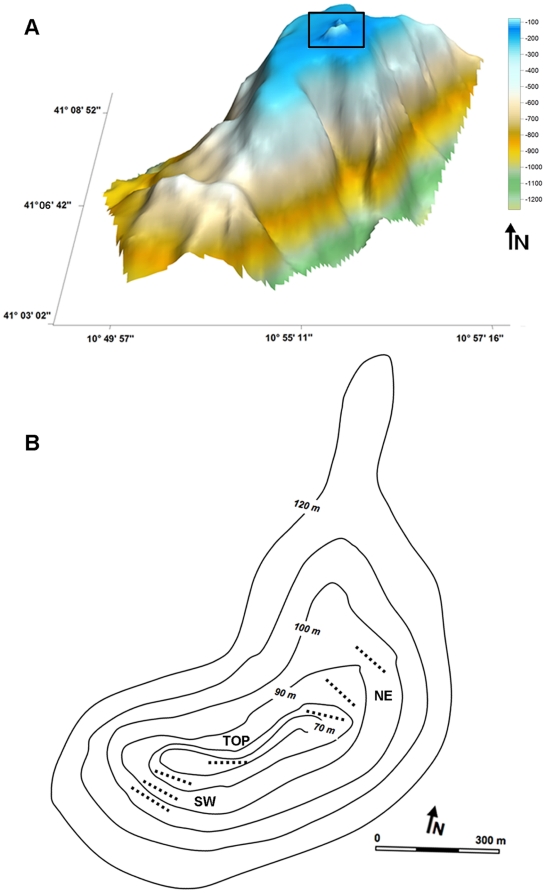
Topography of the Vercelli Seamount. a) Three-dimensional map of the Vercelli Seamount showing on its top the rocky pinnacle explored with the ROV (black rectangle). b) Bathymetric map of the studied peak (60–100 m depth) showing the position of the seven video-transects on the TOP and along the NE and SW flanks.

Vercelli Seamount is a granite intrusion north-west of Sardinia (North Tyrrhenian Sea) that occurred during the Late Miocene [19], [28], [29]. It is the most important part of a complex SW-NE oriented system of ridges arising from a bottom of about 2000 m (Fig. 2a).

The geomorphological characteristics of the seamount have been studied by grabbing, dredging and underwater photoprofiling [19], [30]. These studies showed that the elevation arises from the muddy sea bottom with steep walls (about 20° slope) characterised by isolated granites and clay-limestone concretions emerging from the coarse detrital sand. Around 200–250 m depth, walls turn into flat planes covered by organogenic coarse and medium sand, gently sloping (1°–3° slope) up to about 100 m depth where a rocky peak rises reaching its maximal elevation around 60 m depth (Fig. 2a). This 2 miles elongated pinnacle (41°06.45′N–10°54.43′E, summit of the peak), represents the survey area. Here the bedrock takes the form of ridges separated by joints of various size and extent, traced by sandy sediments and running north eastward [19]. The two flanks of the pinnacle (NE and SW) show a different inclination being steeper on the SW side (17°30′) respect to the NE one (11°) [30].

The exposed granites of the peak, the terrace-like benches of the slopes and the organogenic sand on the plateau are considered consequences of the emersion, and subsequent subsidence, that the pinnacle experienced during the Pliocenic 130 meter glacial-eustatic decrease of the sea level [19], [30]. The seamount has been, and still is, subjected to tectonic movements accompanied by hydrothermal activity responsible for the iron-manganese crust (up to 2 cm thick) found on the deep granites [19].

The mesoscale oceanography of the Tyrrhenian Sea is characterized by a major cyclonic circulation along its boundary, while in the interior several gyre structures can be observed. Vercelli Seamount is located within the transition area between two gyres: cyclonic in the North and anticyclonic in the South [31]. These gyres are usually considered wind-driven [31], [32], but recent investigations revealed the relevant role played by topography [33], [34]. The consequence of this hydrographic situation is the prevalence of upwelling conditions in the northern region and downwelling in the southern one. This almost steady situation is well reproduced both in the surface and in the deep layers. Also temperature and salinity distributions suggest a divergence region in the North and a convergence region in the South. These characteristics are confirmed by the corresponding dissolved oxygen and nutrient distributions [35].

### Benthos data acquisition

Our research has focused on the megabenthic assemblages of the mesophotic shallow peak of the Vercelli Seamount (Fig. 2), where the term mesophotic or *twilight zone* generally refers to the bathymetric belt extended from 40 to 150 m depth, in the lower portion of the photic zone [36]. In this study the rocky mesophotic zone extends up to about 100 m depth, ending in a gently sloping coarse detritic plane.

The distribution of the taxa present in the study area was evaluated on seven horizontal video-transects (each one about 100 m long) recorded at seven depth ranges along the upper rocky portion of the mount (Fig. 2b), by an observer class Remotely Operated Vehicle, ROV *Pluto* (Gaymarine, Switzerland). The depth ranges of the transects were: 60–70 m (the TOP of the seamount), and 70–80 m, 80–90 m and 90–100 m respectively for the NE and SW sides. The 10 m wide depth ranges were selected *a priori* on the base of previous studies concerning distribution patterns of mesophotic benthic organisms [37].

The ROV was equipped with an underwater acoustic tracking position system (HDR made by Gaymarine ultrashort baseline operating with a 30 kHz responder), providing records of its track along the seabed. Additionally it had a depth sensor, a compass, and two parallel laser beams (90° angle) providing a 10 cm scale for measuring the areas of the frames (between 0.5–3 m^2^ each).

The ROV, moving at 1 m height from the seabed (about 2 m of visual field, corresponding to a total of 1400 m^2^ explored area), was equipped with a digital camera (Nikon Coolpix 8700, 8 megapixel) and a high definition video camera (Sony DV 3CCD mod 950). The density (in terms of specimens or colonies m^−2^) or covering (% estimation) of 11 conspicuous and recognizable megabenthic species (or species groups) were evaluated on 14 video frames randomly obtained for each video-transect, for a total of 98 frames. Species considered were: the octocorals *Paramuricea clavata* (Risso, 1826), *Eunicella cavolinii* (Koch, 1887) and *Paralcyonium spinulosum* (Delle Chiaje, 1822), porifera such as *Axinella* spp., *Tethya citrina* Sarà and Melone, 1965, and encrusting sponges, the tube polychaete *Sabella pavonina* (Savigny, 1818), the ascidian *Diazona violacea* (Savigny, 1816), the echinoderms *Echinus melo* Lamarck, 1816 and *Antedon mediterranea* Lamarck, 1816, and the kelp *Laminaria rodriguezii* Bornet 1888. Data were corrected for individual sample areas.

To confirm the taxonomic determination of the specimens counted in the frames, we examined samples collected by dredging (dredge mouth 60 cm in diameter) on the seamount (60–100 m depth) during an oceanographic campaign on board of the R/V *Urania* in May 2009. A more complete list of the recorded species (and their relative abundance) (Table 1) was obtained both by the analysis of the dredged samples and the examination of the whole video and photographic material.

**Table 1:** List of the species and their relative abundance on the peak of the Vercelli Seamount.

Taxa	Relative abundance		
	60–70 m TOP	70–100 m SW	80–100 m NE
**ALGAE**			
cf. *Hydrolithon boreale*		*	
*Laminaria rodriguezii*	***		
**PORIFERA**			
*Axinella damicornis*		**	**
*Axinella polypoides*		*	*
*Axinella verrucosa*		**	**
*Axinyssa aurantiaca*			*
*Ciocalypta penicillus*		*	
*Cliona nigricans*	*		
*Dictyonella incisa*			*
*Dictyonella obtusa*			*
*Dysidea* sp.		*	
*Haliclona* (*Soestella*) *implexa*		*	
*Haliclona* sp.		*	
*Leuconia* sp.		*	
*Myxilla* (*Myxilla*) *rosacea*		*	
*Phorbas fictitius*		*	
*Plakortis simplex*		*	
*Polymastia mamillaris*		*	
*Tethya citrina*	***	**	***
**CNIDARIA**			
Aglaopheniidae g.sp.		*	
*Alcyonium coralloides*		**	
*Alcyonium palmatum*			*
*Amphianthus* sp.		*	
*Antennella* sp.		*	*
*Antipathella subpinnata*		*	
*Callogorgia verticillata*		*	
*Corallium rubrum*		*	
*Corynactis viridis*	*	**	*
*Dendrophyllia cornigera*		*	
*Eunicella cavolinii*		***	**
Haleciidae g.sp.		*	*
*Lafoea dumosa*		*	
Lafoeidae g.sp.		*	*
*Paralcyonium spinulosum*		*	***
*Paramuricea clavata*		***	*
*Paramuricea macrospina*		**	*
*Parazoanthus axinellae*		**	**
*Pelagia noctiluca*		*	
Sertularidae g.sp.		*	
**POLYCHAETA**			
*Filograna* spp.		***	*
*Sabella pavonina*		*	***
Spirorbinae g.sp.	***		
**MOLLUSCA**			
*Chlamys* spp.		*	
*Calliostoma* spp.		*	
*Octopus vulgaris*			*
*Patella* spp.		*	
*Pterya hirundo*		*	
*Solenogastres* g.sp.		*	
**CRUSTACEA**			
*Parthenope macrochelos*		*	
*Rochinia rissoana*		*	
**BRYOZOA**			
*Adeonella* sp.		*	*
*Smittina cervicornis*		*	*
*Turbicellepora avicularis*		*	
**ECHINODERMATA**			
*Antedon mediterranea*	***		
*Astrospartus mediterraneus*		*	
*Cidaris cidaris*		*	*
*Echinaster sepositus*	*		
*Echinus melo*		**	**
*Hacelia attenuata*		*	*
*Holoturia polii*		*	**
*Ophidiaster ophidianus*		*	*
*Ophiotrix* spp.		**	
**TUNICATA**			
*Diazona violacea*		*	***
Didemnidae g.sp.		*	*
**OSTEICHTHYES**			
*Anthias anthias*		**	**
*Coris julis*	***		
*Muraena helena*	*		
*Serranus cabrilla*		*	*
*Trachurus* sp.			*

Symbol legend: * = rare, ** = common, *** = very abundant.

### Statistical analyses

We hypothesized that species density (or covering) and structure of the megabenthic assemblages along the opposite flanks of the Vercelli Seamount differed significantly and that these differences varied with water column depth.

To test our hypothesis, for 6 representative taxa of the flanks separately (*Paramuricea clavata*, *Eunicella cavolinii*, *Paralcyonium spinulosum*, *Axinella* spp., Encrusting sponges, *Sabella pavonina*, *Diazona violacea*), we used a 2-way analysis of variance (ANOVA) with flank (fixed factor with two levels: NE and SW) and water column depth (fixed factor with three levels: 70–80, 80–90 and 90–100 m) as sources of variance, with n = 14 for the combination of factors. Prior to analyses, the homogeneity of variance was tested by means of the Cochran's test and, when necessary, the data were appropriately transformed. For those data sets for which the transformation did not allow to obtain homogeneous variances, a more conservative level of significance was considered [38]. When significant differences were observed, a *post-hoc* Student-Newman-Kuels test (SNK) was also performed. All ANOVA and SNK tests were carried out using the GMAV software (University of Sidney).

To test for differences between the assemblages observed along the two flanks of the Vercelli Seamount at different water column depths, a distance-based permutational multivariate analysis of variance (PERMANOVA, [39], [40]) based on a partial benthos dataset (11 taxa) was used (first design). Such dataset was based on the most representative species recognisable from video sequences. PERMANOVA was also used to test for differences between benthic assemblages at the TOP of the seamount (60–70 m depth) and those in the flank stations located at the closest water column depth (i.e. 70–80 m NE and 70–80 m SW) (second design).

The PERMANOVA test is an analogous to the multivariate analysis of variance (MANOVA), which however is too stringent in its assumptions for most ecological multivariate data sets [39]. Non-parametric methods based on permutation tests such as the one performed by the PERMANOVA tool are preferable since they allow to partition the variability in the data according to a complex design or model and to base the analysis on a multivariate distance measure that is reasonable for ecological data sets [40].

The first design included 2 fixed and orthogonal factors: flank (2 levels: NE vs. SW) and water depth (3 levels: 70–80 m, 80–90 m, and 90–100 m), with n = 14 for combination of factors (a total of 84 video frames, 42 per flank, 14 replicate frames per depth range). The analysis was based on Bray-Curtis similarity on previously presence/absence transformed data, using 999 random permutations of appropriate units, with permutation of residuals under a reduced model [41]. Since the interaction Flank×Depth was found significant, it was further analysed through pairwise comparisons.

The second design included one fixed factor: (3 levels: TOP, NE flank at 70–80 m depth and SW flank at 70–80 m depth), again with n = 14 for combination of factors (a total of 42 video frames, 14 replicate frames per depth range). The analysis was based on Bray-Curtis similarity on previously presence/absence transformed data, using 999 random permutations of appropriate units, with unrestricted permutation of raw data [41]. Pairwise comparisons were also carried out.

Permutational multivariate analysis of variance was run by means of the PERMANOVA routine included in the PRIMER6+ software (Plymouth Marine Laboratory).

To identify the taxa explaining the differences between the different stations, SIMPER analyses were carried out both on presence/absence and square-root transformed data using Bray-Curtis similarity with a 90% cut-off for low contributions. We kept the information provided by the square-root transformation to provide information on the weight of abundant vs. rare species abundance. SIMPER analyses were carried out by means of PRIMER6+ software (Plymouth Marine Laboratory).

## Results

A total of 69 taxa (comprehensive of both megabenthos and ichthyofauna) were identified from the analysis of videos, photos and dredged material (Table 1). Of all taxa, suspension feeders were the most represented, especially cnidarians (20 taxa) and sponges (17 taxa).

**Figure 3:**
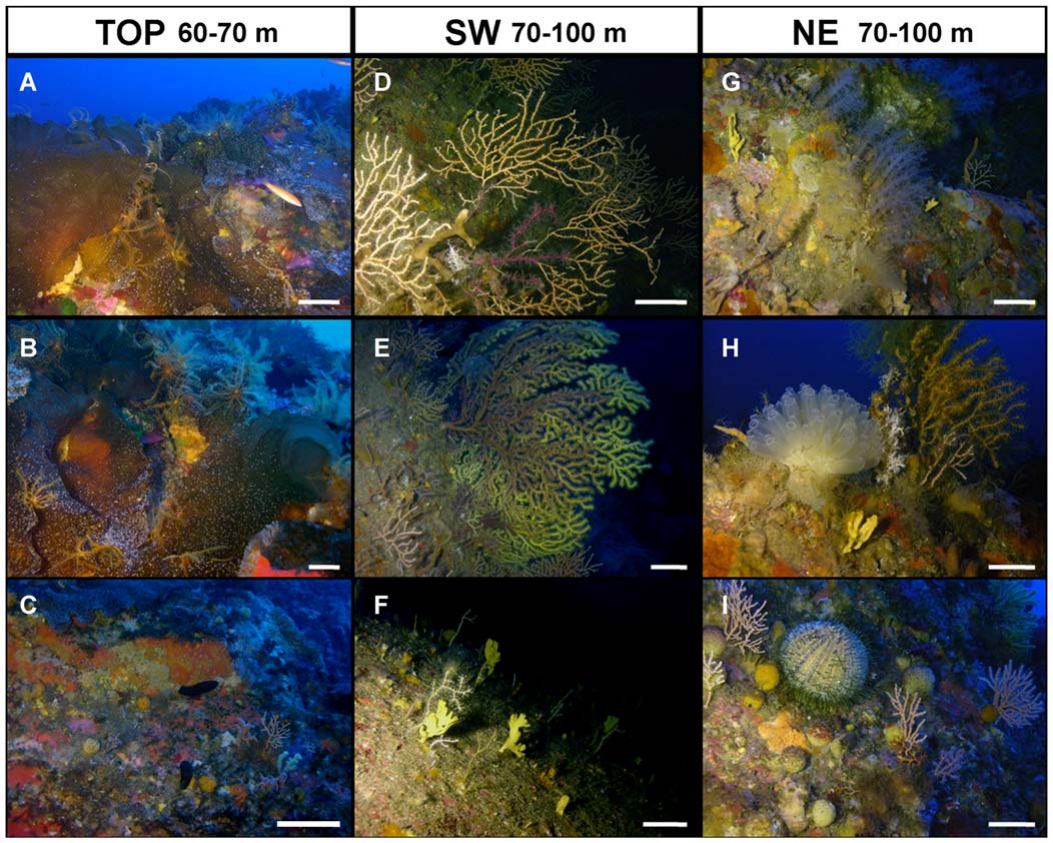
Aspects of the benthic assemblages of the Vercelli Seamount's peak. TOP region (a–c): a) the kelp *Laminaria rodriguezii* lying on the summit pinnacle. b) *Antedon mediterranea* on *L. rodriguezii* c) Encrusting sponges, bryozoans and coralline algae covering the rocky surface. SW region (d–f): d) Colonies of *E. cavolinii* partially covered by the soft coral *Alcyonium coralloides*. e) Large specimens of *P. clavata* together with *Eunicella cavolinii*. f) Several *Axinella* spp. sponges on a sparsely colonised surface. NE region (g–i): g) Group of *Paralcyonium spinulosum* colonies. h) Colony of the ascidian *Diazona violacea*. i) Small sparse colonies of *E. cavolinii* together with numerous *T. citrina* sponges and an *Echinus melo* urchin. Scale bar: 10 cm.

**Figure 4:**
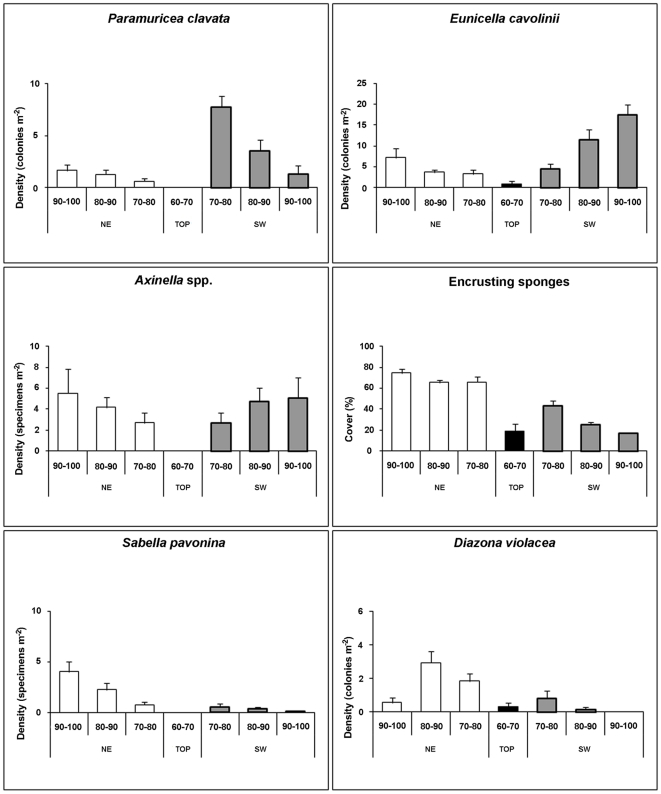
Species distribution. Density (specimens or colonies m^−2^) and covering (%) distribution of six species found on both sides of the peak at the different depth ranges.

The seamount summit showed a dense population of the kelp *Laminaria rodriguezii* (Fig. 3a–b) covering almost 80% of the substratum. The substratum free from the alga was colonised by encrusting sponges (Fig. 3c, 4). Many specimens of the orange crinoid *Antedon mediterranea* (on average 49,7 individuals m^−2^) occurred on the algal thalli together with dense aggregations of undetermined spirorbids (Fig. 3a–b).

The flanks of the Vercelli Seamount were characterised by different bathymetric distributions of the considered taxa (Fig. 4, Table S1). The southern side of the pinnacle was characterised by the dominance of branched octocorals which sometimes reached very high densities. On the rocky boundary close to the detritic bottom (90–100 m) of this flank the only large coral present was the octocoral *Eunicella cavolinii*, with an average density of 17.5 colonies m^−2^ and the more rare *Paramuricea clavata* (on average 1.3 colonies m^−2^) (Fig. 3d–e, 4). Specimens of *Callogorgia verticillata* (Pallas, 1766), *Corallium rubrum* (Linnaeus, 1758) and the scleractinian *Dendrophyllia cornigera* were occasionally observed in the deepest part (90–100 m) of the pinnacle. The only recognisable sponge species in this zone were *Axinella* spp., mainly *Axinella verrucosa* (Esper, 1794) and *Axinella damicornis* (Esper, 1794), with a mean density of about 5.1 specimens m^−2^ (Fig. 3f, 4). The encrusting sponges had an average estimated cover of 17% (Fig. 3f, 4). At this level some dead colonies of *E. cavolinii* were recorded, often covered by the pink parasitic octocoral *Alcyonium coralloides* (Pallas, 1766) (Fig. 3d) or colonized by the tube worm *Sabella pavonina*. Neither traces of predation nor physical damages (both natural and human) were highlighted as causes for the death of the colonies.

In the 80–90 m and 70–80 m depth ranges of the southern flank the density of *P. clavata* progressively increased (up to 7.8 colonies m^−2^) while that of *E. cavolinii* decreased (to 4.5 colonies m^−2^) (Fig. 4). One colony of the arborescent antipatharian *Antipathella subpinnata* (Ellis and Solander, 1786) was observed at about 90 m depth, where encrusting sponges progressively increased their covering up to 43% (Fig. 4).

At all depths, the benthic assemblage settled on the northern side of the seamount was significantly different from that of the southern flank (pairwise comparison after PERMANOVA, P<0.01 for all depths; Table S2). From the bottom to 80 m depth we recorded a very large number of colonies of the blue octocoral *Paralcyonium spinulosum* (up to 4.3 colonies m^−2^) found exclusively on this flank of the seamount (Fig. 3g). The branched yellow *Alcyonium palmatum* (Pallas, 1766) was occasionally observed. *P. clavata* and *E. cavolinii* were also present on the northern side but with densities (1.7 and 7.1 colonies m^−2^ respectively) significantly lower than on the southern side (SNK test, P<0.05; Fig. 4, Table S1). The density of *Axinella* spp. (Fig. 3f, g–h) at all depths was similar on both sides of the seamount (Fig. 4, Table S1), but the cover of encrusting sponges (Fig. 3g) (almost 80%) was significantly higher on the northern flank (SNK test P<0.05; Fig. 4, Table S1). *Tethya citrina* (Fig. 3i) was mainly present up to 80 m depth on both sides, 2.0 and 2.8 specimens m^−2^ respectively for the north and south side. The ascidian *Diazona violacea* (Fig. 3h) and the polychaete *S. pavonina* were extremely abundant (3.0 colonies m^−2^ and 4.0 specimens m^−2^ respectively) on the northern flank while they were significantly less abundant on the southern one (SNK test, p<0.05; Fig. 4, Table S1).

The PERMANOVA test revealed significant differences in the benthic assemblages between the two seamount flanks (PERMANOVA, P<0.001; df = 1). Pairwise comparisons revealed that: i) differences were consistently significant at all sampling depths (P<0.001, for all depths); ii) dissimilarity between NE and SW flanks increased with increasing water depth; iii) in both flanks the largest differences were observed between assemblages at 70–80 m and those at 90–100 m depth after a square-root transformation (Table S2). The SIMPER analysis carried out on the data set from the two flanks revealed that differences between benthic assemblages in the NE and SW flanks were mostly due to the encrusting sponges, which contributed 61%, 55% and 56% at 70–80 m, 80–90 m and 90–100 m depth, respectively. *Eunicella cavolinii* explained a significant proportion of differences between the seamount flanks, but only at 80–90 m (19%) and at 90–100 m (27%).

The PERMANOVA test revealed significant differences in the benthic assemblages also between the TOP and the stations located at the closest depth range in the two different flanks (P<0.001, df = 2; Table S3i). The pairwise comparisons carried out on this reduced data set (i.e. TOP vs. flank stations at 70–80 m depth) revealed that the assemblage on the TOP differed from that of the SW flank more than from that of the NE flank (Table S3ii). The SIMPER analysis based on a square-root transformation revealed that the differences between the summit and the NE and SW flanks, respectively, were mostly due to *Laminaria rodriguezii* (29% and 32%, respectively), *Antedon mediterranea* (23% both) and encrusting sponges (23% and 16%).

## Discussion

In this study we describe, for the first time, the species composition, observed through ROV imaging, of the mesophotic megabenthic assemblages of a Tyrrhenian seamount.

The rocky pinnacle of the Vercelli Seamount hosts a notable community characterised by high species richness and high abundances. This result is consistent with studies conducted both in the Atlantic and Pacific oceans, showing the highest aggregations of suspension feeders near the peaks of seamounts, especially in relation to strong currents thus low sedimentation rates [10], [42], [43]. In particular in the case of Vercelli, the presence of a rich benthic community could be related to the substrate availability and the hydrodynamic conditions of the area [31], [32] characterised by upwelling conditions likely producing re-suspension of organic particles favouring suspension feeding organisms [35].

In case of shallow rocky mounts this abundance is also related to the fact that the peaks, far from sources of terrigenous turbidity, penetrate in the euphotic zone allowing the massive development of algae (also at great depths) and associated faunal assemblages [2]. From this point of view, Vercelli Seamount resembles other shallow seamounts in oceanic basins, often hosting kelp canopies. For example the area of the Gorringe Bank (minimum depth less than 50 m) in front to the Strait of Gibraltar is characterised by a dense assemblage of *Paramuricea clavata* and, on its upper portion, by a dense meadow of *Laminaria ochroleuca* De La Pylaie, 1824 [44]. A kelp forest of *Ecklonia biruncinata* (Papenfuss, 1944) is found on the summit of Vema Seamount (summit less than 40 m depth), in the South Atlantic Ocean [45], [46]. As for the other meadows, also the one composed of *Laminaria rodriguezii* occurring on the summit of the pinnacle of the Vercelli Seamount, is characterised by phylloids covering almost horizontally the rocky bottom and enhancing in this way light capture.

Rich benthic assemblages were recorded also on Pacific shallow water seamounts. The summit of Cobb Seamount (about 24 m depth), for example, is covered by encrusting algae and a dense population of rock scallops, which, in the mesophotic zone, are used as secondary substrates for a dense assemblage of suspension feeders [47], [48]. Similarly to Vercelli, Bowie Seamount (summit about 24 m depth) hosts a dense kelp canopy on the top of its rocky peak [49].

The shallow Mediterranean underwater mountains share common characteristics as the presence of an apical coralligenous assemblage, the typical biocoenosis growing on calcareous formations of biogenic origin, produced by the accumulation of encrusting algae in dim light conditions. The depth range at which this assemblage is found depends on the amount of irradiance reaching the sea bed and may vary from 20 m to 120 m [50]. Our observations on the Vercelli are similar to those made on the other four shallow Mediterranean seamounts (peaks between 80 and 100 m depth) so far explored with the aid of a ROV, and located in the Alboran Sea, the Mallorca Channel and off Murcia [27]. A part from various coralligenous formations, maërl beds were also detected on these structures. Various species of suspension feeders and detritivorous species are listed, but no specific distribution of the organisms is given [27]. Similarly a coralligenous biocoenosis has been found also on the top of the Amendolara Bank in Ionian Sea [24].

Recent studies have indicated that seamounts may not show high endemism rates [5], [51], as previously suggested [52]–[54], but they may host a variety of communities or species that are rarely encountered, or are represented by a very low density on slope habitats [6], [55]–[57]. This is the case of Vercelli Seamount where some species were encountered with a remarkably high abundance respect to Tyrrhenian coastal areas. The Atlantic brown alga *L. rodriguezii*, known only for some localities of the Mediterranean Sea, is adapted to mesophotic areas, with temperatures less than 15°C [58]. A well known canopy of this algae has been reported only for the rocky summit (around 40 m depth) of Apollo's Bank (Ustica Island, Sicily) [59]. Such rocky habitats are important since the records of *L. rodriguezii* for the rhodolith beds [60] have suffered more the trawling impact [50]. This kelp shows strong seasonal variations of biomass with rapid growth in spring and sorus formation in summer to autumn [61]. The summer growth of *L. rodriguezii* is visible also on the seamount (Fig. 3a–b), as the algal sheets cover encrusting sponges or small living octocorals visible underneath. In autumn, the decaying of the distal part of the phylloids produces a relevant supply of organic biomass for several organisms such as sea urchins (*Echinus melo*, *Cidaris cidaris*) living on the detritic bottoms surrounding the pinnacle. The trophic contribution of kelp debris to deep communities has been well studied in several coastal environments [e.g. 62], but no information is present for shallow seamount ecosystems. Kelp debris processed by heterotrophic prokaryotes is typically enriched in nitrogen [62] thus representing a high-energy source of food for benthic consumers in coastal environments [63], [64], [65]. Since deep-sea ecosystems depend largely upon the organic matter sinking from the upper water column layers, the sinking of kelps thalli might represent an important fuel also for the seamount's deep food chains dominated by grazers, such as sea urchins [66], gastropods [62], and detritivorous organisms such as amphipods [62] or bivalves [67].

Also the ascidian *Diazona violacea* (Fig. 3h), a typical circalittoral Atlanto-Mediterranean species, is characterised by a strong seasonality. This species reproduces sexually in summer while during the cold season undergoes bud formation, regenerating complete new zooids only in the next spring [68]. The presence of seasonal species, which disappear during a part of the year, is a typical element of rich and diverse habitats, hosting a complex biodiversity varying not only in space, but also in time [69].

Particularly interesting is the unusually large population of the sponge *Tethya citrina* (up to 6 specimens m^−2^ on the northern flank) (Fig. 3i). In the Mediterranean Sea, this species is known primarily from areas characterised by high sedimentation rates, like lagoons or muddy fiords [70], and it is only sporadically recorded for coralligenous habitats (Bavestrello, unpublished data). This is the largest Mediterranean population ever recorded and the presence of this habitat type could be an indicator of light turbulence regime and higher sedimentation levels on the northern side of the seamount.

Another remarkable component of the assemblage of the northern flank is the soft coral *Paralcyonium spinulosum* (Fig. 3g), which is generally sparse and rarer than the other Mediterranean species of alcyonaceans [71]. This species has been recently described with lower abundances for high sedimentation zones like the North Adriatic concretions [71] or the muddy mesophotic bottom of the Gulf of S. Eufemia along the Calabrian coast [37], [72].

Branched octocorals such as *P. clavata* and *Eunicella cavolinii* are major components of the shallow and mesophotic rocky environments of the Mediterranean Sea, where they show a bathymetric distribution very similar to that observed on Vercelli Seamount [37]. Very dense forests of these anthozoans, especially of *P. clavata*, have been recorded along the rocky Tyrrhenian coastline, from the northern basin (up to 70 colonies m^−2^) [73] to the southern one (up to 26 colonies m^−2^) [74].

These examples support the idea that the conservation value of the Vercelli Seamount shouldn't be focused mainly on endemism, but on the variety of communities that this system supports. These assemblages moreover may act as source populations of larvae for neighbouring coastal ecosystems particularly in those Tyrrhenian areas which recently suffered extended mass mortality events of benthic organisms [75].

From this point of view the pinnacle of the Vercelli Seamount hosts three notable assemblages developing in a relatively limited space. *Laminaria rodriguezii*, the numerous crinoids *Antedon mediterranea* living on the kelp and the encrusting sponges covering the rocky surface of the summit underneath the algal sheets, differentiate the first 10 m of the pinnacle from the closest depth ranges of both flanks. These are characterized by a steeper inclination more suitable for erected organisms, such as massive sponges and corals, depending on the water movement for their feeding activity. The northern 70–80 m depth range results more similar to the TOP respect to the southern side because of the high maximal density of *T. citrina* and the wide cover of encrusting sponges characterizing both stations. In the 70–80 m depth range on the southern flank instead *P. clavata* is already the dominant species and the dissimilarity with the summit is higher (Table S3ii).

As shown by the statistical analysis, the two flanks of the pinnacle of the Vercelli Seamount host different assemblages (Table S2). The southern side is dominated by a wide development of organisms, mainly passive filter-feeders such as octocorals, adapted to live in environments characterised by high hydrodynamic conditions [37]. The species composition of this assemblage varied also according to depth. In particular *P. clavata* and *E. cavolinii* show an opposite trend of bathymetric distribution, with *E. cavolinii* being distributed preferentially in the deepest ranges, as already observed for other mixed octocoral forests of the Mediterranean Sea [37].

The northern side, on the contrary, is mainly dominated by active filter-feeders like sponges, polychaetes and ascidians adapted to live in habitat characterised by lower hydrodynamic conditions.

Encrusting sponges and *E. cavolinii* mainly contribute in the separation of the flanks assemblages, being the most abundant taxa, in term of percent covering or density. Their major contribution is in agreement with their bathymetric abundance distribution. The PERMANOVA analysis evidenced an increasing dissimilarity (based on square root transformation) of the benthic assemblages along the two flanks, according to water column depth. This result suggests that there is an effect of depth on the β-diversity of the seamount megabenthic community, being the deepest assemblages the most dissimilar. These observations confirm that seamounts, showing high habitat heterogeneity, are able to host different assemblages characterised by different ecological requirements [76], [77].

The observed differences between the megafauna assemblages recorded on the two flanks of the pinnacle, both in term of composition and trophic strategy, are probably related to local current conditions. Hydrodynamism most likely is stronger along the southern flank respect the northern one. This situation, together with the lower inclination of the slope, probably increases the sedimentation rates on the northern flank dominated by active suspension feeders.

The Vercelli Seamount is not heavily exploited by professional fishing. Nevertheless, some abandoned nets and lines were observed along the ROV track. Environments characterised by high biodiversity should be worthy of protection by international conservation programs as already suggested for others deep diversity oases of the Mediterranean Sea [37], [72].

## Supporting Information

Table S1 ANOVA. Output of the 2-ways ANOVA testing for differences in the density of the different megabenthic taxa among the two flanks of the Vercelli Seamount's peak with water column depth. Reported are also the results of SNK pairwise comparisons.
(DOC)

Table S2 PERMANOVA first design. i. Results of the PERMANOVA testing for differences in benthic assemblages between the two investigated flanks of the Vercelli Seamount's peak at different water column depths. ii–iii. Results of post-hoc pairwise comparisons and the results of the SIMPER analysis (average dissimilarity) for both NE vs. SW (ii) and depth ranges comparisons (iii).
(DOC)

Table S3 PERMANOVA second design. i. Results of the PERMANOVA testing for differences in benthic assemblages between the top seamount (60–70 m depth) and the two flank stations located at the closest water column depth (i.e., 70–80 m depth). ii. Results of the SIMPER analysis (average dissimilarity).
(DOC)

## References

[1] Pitcher TJ, Morato T, Hart PJB, Clark MR, Haggan N (2007). Seamounts: Ecology, Fisheries & Conservation.

[2] Boehlert GW, Genin A, Keating BH, Fryer P, Batiza R, Boehlert GW (1987). A review of the effects of seamounts on biological processes.. Geophys Monogr Ser.

[3] Rogers AD (1994). The biology of seamounts.. Adv Mar Biol.

[4] Genin A, Dower JF, Pitcher TJ (2007). Seamount plankton dynamics.. Seamounts: Ecology, Fisheries & Conservation.

[5] Clark MR, Rowden AA, Schlacher T, Williams A, Consalvey M (2010). The Ecology of Seamounts: Structure, Function, and Human Impacts.. Annu Rev Mar Sci.

[6] Samadi S, Schlacher T, Richer de Forges B, Pitcher TJ (2007). Seamount benthos.. Seamounts: Ecology, Fisheries & Conservation.

[7] Lundsten L, Barry JP, Cailliet GM, Clague DA, DeVogelaere AP (2009). Benthic invertebrate communities on three seamounts off southern and central California, USA.. Mar Ecol Progr Ser.

[8] Genin A (2004). Bio-physical coupling in the formation of zooplankton and fish aggregations over abrupt topographies.. J Mar Syst.

[9] Carney RS (2005). Zonation of deep biota on continental margins.. Oceanogr Mar Biol Annu Rev.

[10] Genin A, Dayton PK, Lonsdale PF, Spiess FN (1986). Corals on seamount peaks provide evidence of current acceleration over deep-sea topography.. Nature.

[11] Genin A, Paull CK, Dillon WP (1992). Anomalous abundances of deep-sea fauna on a rocky bottom exposed to strong currents.. Deep-Sea Res.

[12] Matsumoto AK (2010). Estimation of *in situ* distribution of carbonate produced from cold-water octocorals on a Japanese seamount in the NW Pacific.. Mar Ecol Prog Ser.

[13] Kitchingman A, Lai S, Morato T, Paulay D, Pitcher TJ (2007). “How many seamounts are there and where are they located?”.. Seamounts: Ecology, Fisheries & Conservation.

[14] Genesseaux M, Rehault JP, Thomas B, Colantoni P, Fabbri A (1986). Résultats de plongées en submersible CYANA sur les blocs continentaux basculés et le volcan Vavilov (Mer Tyrrhénienne centrale).. C R Acad Sci Paris.

[15] Robin C, Genesseaux M, Colantoni P, Vanney JR (1986). Le volcan sous-marin quaternaire Vavilov (mer Tyrrhénienne centrale). Résultats de plongées en submersible CYANA.. C R Acad Sci Paris.

[16] Uchupi E, Ballard RD (1989). Evidence of hydrothermal activity on Marsili Seamount Tyrrhenian Basin.. Deep-Sea Res I.

[17] Varnavas SP, Papaioannou J, Catani J (1988). A hydrothermal manganese deposit from the Eratosthenes Seamount Eastern Mediterranean Sea.. Mar Geol.

[18] Zhuleva EV (1988). Submarine photoprofiling in a geological study of the Vercelli Seamount (Tyrrhenian Sea).. Oceanol.

[19] Galil B, Zibrowius H (1998). First Benthos Samples From Eratosthenes Seamount Eastern Mediterranean.. Sencken Marit.

[20] UNEP (2003). The “white coral community”, canyon and seamount faunas of the deep Mediterranean Sea: distribution, biological richness and interest of the white coral community: Project for the Implementation of a Strategic Action Plan for the Conservation of Biological Diversity in the Mediterranean (SAP BIO): Regional Documents prepared within the framework of the SAPBIO Project..

[21] Panetta P, Dell'Angelo B, Fiordiponti F (1985). I Poliplacofori Del Banco Dell'Amendolara (Golfo Di Taranto).. Oebalia.

[22] Perrone A (1985). Report on the biological survey of Amendolara Seamount: Nudibranchia of Amendolara Seamount.. J Mollus Stu.

[23] Strusi A, Tursi A, Cecere E, Montanaro C, Panetta P (1985). The Amendolara Seamount (High Ionian Sea): General Description.. Oebalia.

[24] Cecere E, Perrone C (1988). First Contribution to the Knowledge of Macrobenthic Flora of the Amendolara Sea-Mount (Ionian Sea).. Oebalia.

[25] Blanc JJ (1964). Campagne de la Calypso en Méditerranée nord-orientale (1960). 5.Recherches géologiques et sédimentologiques.. Ann Inst oceanogr Paris.

[26] Blanc JJ, Froget C (1967). Campagne de la Calypso en Méditerranée orientale (1964). 1.Recherches de géologie marine et sédimentologie.. Ann Inst oceanogr Paris.

[27] Aguiliar R, Pastor X, de la Torriente A, García S, Pergent-Martini C, Brichet M (2009). Deep-sea coralligenous beds observed with ROV on four seamounts in the Western Mediterranean.. UNEP-MAP-RAC/SPA, Proceedings of the 1^st^ Mediterranean symposium on the conservation of the coralligenous and others calcareous bio-concretions.

[28] Barberi F, Bizouard H, Capaldi G, Hsu K, Montadert L (1978). Age and nature of basalts from Tyrrhenian abyssal plain.. Initial Reports of the DSDP, 42/I.

[29] Barberi M, Gasparotto G, Lucchini F, Savelli C, Vigliotti L (1989). Contributo allo studio del magmatismo nel Mar Tirreno: l'intrusione granitica tardo-Miocenica del monte submarino Vercelli.. Mem Soc Geol It.

[30] Gallignani P (1973). I sedimenti della cima del Monte Vercelli.. Gior Geo.

[31] Artale V, Astraldi M, Buffoni G, Gasparini GP (1994). Seasonal variability of gyre-scale circulation in the northern Tyrrhenian Sea.. J Geophys Res.

[32] Nair R, Cattini E, Gasparini GP, Rossi G (1994). Circolazione ciclonica e distribuzione dei nutrienti nel Tirreno settentrionale..

[33] Budillon G, Gasparini GP, Schroeder K (2009). Persistence of an Eddy Signature in the Central Tyrrhenian Basin.. Deep-Sea Res II.

[34] Vetrano A, Napolitano E, Iacono R, Schroeder K, Gasparini GP (2010). Tyrrhenian Sea Circulation and water mass fluxes in Spring 2004: observations and models results.. J Geophys Res.

[35] MEDATLAS (2002). http://www.ifremer.fr/medar/.

[36] Lesser MP, Slattery M, Leichter JJ (2009). Ecology of mesophotic coral reefs.. J Exp Mar Biol Ecol.

[37] Bo M, Bavestrello G, Canese S, Giusti M, Salvati E (2009). Characteristics of a black coral meadow in the twilight zone of the central Mediterranean Sea.. Mar Ecol Prog Ser.

[38] Underwood AJ (1991). Beyond BACI: experimental designs for detecting human environmental impacts on temporal variations in natural populations.. Aust J Mar Freshw Res.

[39] Anderson MJ (2001). A new method for non-parametric multivariate analysis of variance.. Aust Ecol.

[40] McArdle BH, Anderson MJ (2001). Fitting multivariate models to community data: a comment on distance-based redundancy analysis.. Ecol.

[41] Anderson MJ, ter Braak CJF (2003). Permutation tests for multifactorial analysis of variance.. J Stat Comp Simul.

[42] Piepenburg D, Müller B (2004). Distribution of epibenthic communities on the Great Meteor Seamount (North-east Atlantic) mirrors pelagic processes.. Arch Fisch Meeresforsch.

[43] Matsumoto AK, Freiwald A, Roberts JM (2005). Recent observations on the distribution of deep-sea coral communities on the Shiribeshi Seamount, Sea of Japan.. Cold-water corals and ecosystems.

[44] OCEANA (2005). The seamounts of the Gorringe Bank.. http://www.training.oceana.org.

[45] Berrisford CD (1969). Biology and zoogeography of Vema Seamount: a report on the first biological collection made on the summit.. Trans R Soc S Afr.

[46] Kensley B (1980). Decapod and isopod crustaceans from the West coast of Southern Africa, including seamounts Vema and Tripp.. Ann S Afr Mus.

[47] Birkeland C (1971). Biological observations on Cobb Seamount.. Northwest Science.

[48] Parker T, Tunnicliffe V (1994). Dispersal Strategies of the Biota on an Oceanic Seamount: Implications for Ecology and Biogeography.. Biol Bull.

[49] McDaniel N, Swanston D, Haight R, Reid D, Grant G (2003). Biological Observations at Bowie Seamount..

[50] Ballesteros E (2006). Mediterranean coralligenous assemblages: a synthesis of present knowledge.. Ocean Mar Biol.

[51] McClain GR (2007). Seamounts: identity crisis or split personality?. J Biogeog.

[52] Wilson RR, Kaufmann RS, Keating BH, Fryer P, Batiza R, Boehlert GW (1987). Seamount biota and biogeography.. Geophys Monograph.

[53] Parin NV, Mironov AN, Nesis KN (1997). Biology of the Nazca and Sala y Gomez submarine ridges, an outpost of the Indo-West Pacific fauna in the eastern Pacific Ocean: composition and distribution of the fauna, its communities and history.. Adv Mar Biol.

[54] Richer de Forges B, Koslow JA, Poore GCB (2000). Diversity and endemism of the benthic seamount fauna in the southwest Pacific.. Nature.

[55] Hall-Spencer JM, Rogers A, Davies J, Foggo A, George RY, Cairns SD (2007). Historical deep-sea coral distribution on seamount, oceanic island and continental shelf-slope habitats in the NE Atlantic.. Conservation and adaptive management of seamount and deep-sea coral ecosystems.

[56] Thoma JN, Pante E, Brugler MR, France SC (2009). Deep-Sea octocorals and antipatharians show no evidence of seamount-scale endemism in the NW Atlantic.. Mar Ecol Progr Ser.

[57] McClain GR, Lundsten L, Ream R, Barry J, DeVogelaere A (2009). Endemicity, Biogeography, Composition and Community Structure on a Northeast Pacific Seamount.. PLoS ONE.

[58] Giaccone G, Di Martino V (1997). Syntaxonomic relationship of the Mediterranean phytobenthos assemblages: paleoclimatic bases and evolutive tendencies.. Lagascalia.

[59] Giaccone G (1967). Popolamenti a *Laminaria rodriguezii* Bornet sul Banco Apollo dell'isola di Ustica (Mar Tirreno).. Nova Thalassia.

[60] Massutí E, Reñones O (2005). Demersal resource assemblages in the trawl fishing grounds off the Balearic Islands (western Mediterranean).. Sci Mar.

[61] Lüning K, Srivastava LM (1982). Seasonality in larger brown algae and its possible regulation by the environment.. Synthetic and Degradative Processes in Marine Macrophytes.

[62] Norderhaug KM, Fredriksen S, Nygaard K (2003). Trophic importance of *Laminaria hyperborea* to kelp forest consumers and the importance of bacterial degradation to food quality.. Mar Ecol Prog Ser.

[63] Tzetlin AB, Mokievsky VO, Melnikov AN, Saphonov MV, Simdyanov TG, Ivanov IE (2007). Fauna associated with detached kelp in different types of subtidal habitats of theWhite Sea.. Hydrobiol.

[64] Bernardino AF, Smith CR, Baco A, Altamira I, Sumida PYG (2010). Macrofaunal succession in sediments around kelp and wood falls in the deep NE Pacific and community overlap with other reducing habitats.. Deep-Sea Res I.

[65] Schaal G, Riera P, Leroux C (2010). Trophic ecology in a Northern Brittany (Batz Island, France) kelp (*Laminaria digitata*) forest, as investigated through stable isotopes and chemical assays.. J Sea Res.

[66] Bedford AP, Moore PG (1984). Macrofaunal involvement in the sublittoral decay of kelp debris: the detritivore community and species interactions.. Estuar coast Shelf Sci.

[67] Duggins DO, Eckman JE (1997). Is kelp detritus a good food for suspension feeders? Effects of kelp species, age and secondary metabolites.. Mar Biol.

[68] Berrill NJ (1948). The development, morphology and budding of the ascidian *Diazona*.. J Mar Biol Ass UK.

[69] Coma R, Ribes M, Gili JM, Zabala M (2000). Seasonality in coastal benthic ecosystems.. Trends Ecol Evol.

[70] Sarà M, Vacelet J, Boury-Esnault N (1987). A study of the genus *Tethya* (Porifera, Demospongiae) and new perspectives in sponge systematic.. Taxonomy of Porifera, NATO ASI Series.

[71] Fava F, Ponti M (2007). Geographical distribution of *Masella edwardsi* and *Paralcyonium spinulosum* (Octocorallia: Paralcyoniidae).. Biol Mar Medit.

[72] Bo M, Bavestrello G, Canese S, Giusti M, Angiolillo (2010). Coral assemblages off the Calabrian Coast (South Italy) with new observations on living colonies of *Antipathes dichotoma*.. It J Zool.

[73] Harmelin JG, Marinopoulos J (1994). Population structure and partial mortality of the gorgonian *Paramuricea clavata* in the north-western Mediterranean.. Mar Life.

[74] Mistri M, Ceccherelli VU (1996). Effects of a mucilage event on the Mediterranean gorgonian *Paramuricea clavata*. I - Short term impacts at the population and colony levels.. It J Zool.

[75] Cerrano C, Bavestrello G, Bianchi CN, Cattaneo-Vietti (2000). A catastrophic mass-mortality episode of gorgonians and other organisms in the Ligurian Sea (north-western Mediterranean), summer 1999.. Ecol Lett.

[76] Raymore PA (1982). Photographic investigations on three seamounts in the Gulf of Alaska.. Pac Sci.

[77] Koslow JA (1997). Seamounts and the ecology of deep-sea fisheries.. Amer Sci.

