# Supplementary material for: Characteristics of the Mesophotic Megabenthic Assemblages of the Vercelli Seamount (North Tyrrhenian Sea)
Source: PLoS One. 2011 Feb 3;6(2):e16357. doi: 10.1371/journal.pone.0016357 (PMC3033400; doi:10.1371/journal.pone.0016357)
Supplement: Table S1 — ANOVA. Output of the 2-ways ANOVA testing for differences in the density of the different megabenthic taxa among the two flanks of the Vercelli Seamount's peak with water column depth. Reported are also the results of SNK pairwise comparisons. (DOC) [file pone.0016357.s001.doc]

| **2-ways ANOVA** | | | | | | | **Cochran's test** | | | **SNK test** | | | | |
| --- | --- | --- | --- | --- | --- | --- | --- | --- | --- | --- | --- | --- | --- | --- |
|  |  |  |  |  |  |  |  |  |  | Flank | | | Depth | |
| Taxon | Source | SS | DF | MS | F | P | Transformation | C | P | 70-80 | 80-90 | 90-100 | NE | SW |
| *Paramuricea clavata* | Flank | 7,80 | 1 | 7,80 | 0,43 | *** | Ln(X+1) | 0,3024 | ns | NE<SW | ns | ns | 70-80=80-90=90-100 | 70-80>[80-90=90-100] |
|  | Depth | 5,74 | 2 | 2,87 | 12,43 | ** |  |  |  |  |  |  |  |  |
|  | Flank x Depth | 13,95 | 2 | 6,98 | 10,33 | *** |  |  |  |  |  |  |  |  |
|  | Residual | 40,47 | 78 | 0,52 |  |  |  |  |  |  |  |  |  |  |
|  | Total | 67,96 | 83 |  |  |  |  |  |  |  |  |  |  |  |
| *Eunicella cavolinii* | Flank | 880,76 | 1 | 880,76 | 20,06 | *** | Ln(X+1) | 0,3111 | ns | ns | NE<SW | NE<SW | [70-80=80-90]<90-100 | 70-80<80-90<90-100 |
|  | Depth | 972,60 | 2 | 486,30 | 11,08 | *** |  |  |  |  |  |  |  |  |
|  | Flank x Depth | 306,02 | 2 | 153,01 | 3,49 | ns |  |  |  |  |  |  |  |  |
|  | Residual | 3.424,43 | 78 | 43,90 |  |  |  |  |  |  |  |  |  |  |
|  | Total | 5.583,81 | 83 |  |  |  |  |  |  |  |  |  |  |  |
| *Axinella* spp. | Flank | 0,05 | 1 | 0,05 | 0,05 | ns | Ln(X+1) | 0,2492 | ns | na | na | na | na | ns |
|  | Depth | 4,52 | 2 | 2,26 | 2,21 | ns |  |  |  |  |  |  |  |  |
|  | Flank x Depth | 0,26 | 2 | 0,13 | 0,13 | ns |  |  |  |  |  |  |  |  |
|  | Residual | 79,68 | 78 | 1,02 |  |  |  |  |  |  |  |  |  |  |
|  | Total | 84,51 | 83 |  |  |  |  |  |  |  |  |  |  |  |
| Encrusting sponges | Flank | 34.202,68 | 1 | 34.202,68 | 179,41 | *** | No | 0,3824 | ** | NE > SW | NE > SW | NE > SW | 70-80=80-90=90-100 | 70-80>[80-90=90-100] |
|  | Depth | 1.430,95 | 2 | 715,48 | 3,75 | * |  |  |  |  |  |  |  |  |
|  | Flank x Depth | 4.114,29 | 2 | 2.057,14 | 10,79 | *** |  |  |  |  |  |  |  |  |
|  | Residual | 14.869,64 | 78 | 190,64 |  |  |  |  |  |  |  |  |  |  |
|  | Total | 54.617,56 | 83 |  |  |  |  |  |  |  |  |  |  |  |
| *Sabella pavonina* | Flank | 90,11 | 1 | 90,11 | 21,51 | *** | No | 0,5814 | ** | ns | NE > SW | NE > SW | na | na |
|  | Depth | 25,81 | 2 | 12,90 | 3,08 | ns |  |  |  |  |  |  |  |  |
|  | Flank x Depth | 44,86 | 2 | 22,43 | 5,35 | ** |  |  |  |  |  |  |  |  |
|  | Residual | 326,79 | 78 | 4,19 |  |  |  |  |  |  |  |  |  |  |
|  | Total | 487,56 | 83 |  |  |  |  |  |  |  |  |  |  |  |
| *Diazona violacea* | Flank | 48,76 | 1 | 48,76 | 21,05 | *** | No | 0,5032 | ** | NE > SW | NE > SW | NE > SW | [70-80=80-90]>90-100 | 70-80>80-90>90-100 |
|  | Depth | 28,17 | 2 | 14,08 | 6,08 | ** |  |  |  |  |  |  |  |  |
|  | Flank x Depth | 21,60 | 2 | 10,80 | 4,66 | ns |  |  |  |  |  |  |  |  |
|  | Residual | 180,71 | 78 | 2,32 |  |  |  |  |  |  |  |  |  |  |
|  | Total | 279,24 | 83 |  |  |  |  |  |  |  |  |  |  |  |

Table S1. Legend: SS = sum of squares; DF = degree of freedom; MS = mean squares; *** P<0.001, ** P<0.01, * P<0.05; ns = not significant; na = not applicable
