# Supplementary material for: Characteristics of the Mesophotic Megabenthic Assemblages of the Vercelli Seamount (North Tyrrhenian Sea)
Source: PLoS One. 2011 Feb 3;6(2):e16357. doi: 10.1371/journal.pone.0016357 (PMC3033400; doi:10.1371/journal.pone.0016357)
Supplement: Table S2 — PERMANOVA first design. i. Results of the PERMANOVA testing for differences in benthic assemblages between the two investigated flanks of the Vercelli Seamount's peak at different water column depths. ii–iii. Results of post-hoc pairwise comparisons and the results of the SIMPER analysis (average dissimilarity) for both NE vs. SW (ii) and depth ranges comparisons (iii). (DOC) [file pone.0016357.s002.doc]

| **Source** | **df** | **MS** | **Pseudo-F** | **P** |
| --- | --- | --- | --- | --- |
| Flank | 1 | 11805 | 25.3 | *** |
| Depth | 2 | 6427 | 13.8 | *** |
| Flank × Depth | 2 | 2949 | 6.3 | *** |
| Residual | 78 | 468 |  |  |
| Total | 83 |  |  |  |

Table S2i. Symbol legend: *** = P<0.001; ** = p<0.01; * = p<0.05

| **Comparison** | **Depth range (m)** | **t** | **P** | **Average dissimilarity** |
| --- | --- | --- | --- | --- |
| NE vs. SW | 70-80 | 2.572 | ** | 44% |
|  | 80-90 | 4.096 | *** | 52% |
|  | 90-100 | 4.3709 | *** | 67% |

Table S2ii. Symbol legend: *** = P<0.001; ** = p<0.01; * = p<0.05

| **Comparison** | **Flank** | **t** | **P** | **Average dissimilarity** |
| --- | --- | --- | --- | --- |
| 70-80 vs. 80-90 | NE | 2.255 | ** | 28%  31%  22%  43%  55%  37% |
| 70-80 vs. 90-100 |  | 4.194 | *** |
| 80-90 vs. 90-100 |  | 2.759 | *** |
| 70-80 vs. 80-90 | SW | 3.034 | *** |
| 70-80 vs. 90-100 |  | 3.860 | *** |
| 80-90 vs. 90-100 |  | 2.114 | * |

Table S2iii. Symbol legend: *** = P<0.001; ** = p<0.01; * = p<0.05
