# Supplementary material for: Characteristics of the Mesophotic Megabenthic Assemblages of the Vercelli Seamount (North Tyrrhenian Sea)
Source: PLoS One. 2011 Feb 3;6(2):e16357. doi: 10.1371/journal.pone.0016357 (PMC3033400; doi:10.1371/journal.pone.0016357)
Supplement: Table S3 — PERMANOVA second design. i. Results of the PERMANOVA testing for differences in benthic assemblages between the top seamount (60–70 m depth) and the two flank stations located at the closest water column depth (i.e., 70–80 m depth). ii. Results of the SIMPER analysis (average dissimilarity). (DOC) [file pone.0016357.s003.doc]

| **Source** | **df** | **MS** | **Pseudo-F** | **P** |
| --- | --- | --- | --- | --- |
| Site | 2 | 20915 | 37.419 | *** |
| Residual | 39 | 558.9 |  |  |
| Total | 41 |  |  |  |

Table S3i. Symbol Legend: *** = P<0.001; ** = P<0.01

| **Comparison** | **t** | **p** | **Average dissimilarity** |
| --- | --- | --- | --- |
| Top vs. NE flank (70-80m) | 5.2817 | *** | 66%  82%  42% |
| Top vs. SW flank (70-80m) | 10.464 | *** |
| NE flank vs. SW flank | 2.5721 | ** |

Table S3ii. Symbol Legend: *** = P<0.001; ** = P<0.01
